# Dynamic microbial succession of Shanxi aged vinegar and its correlation with flavor metabolites during different stages of acetic acid fermentation

**DOI:** 10.1038/s41598-018-26787-6

**Published:** 2018-06-05

**Authors:** Yunping Zhu, Feifei Zhang, Chengnan Zhang, Li Yang, Guangsen Fan, Youqiang Xu, Baoguo Sun, Xiuting Li

**Affiliations:** 1 Beijing Advanced Innovation Center for Food Nutrition and Human Health, Beijing Technology and Business University (BTBU), Beijing, 100048 China; 2 School of Food and Chemical Engineering, Beijing Technology and Business University, No.33, Fucheng Road, Beijing, 100048 China; 3 Beijing Engineering and Technology Research Center of Food Additives, Beijing Technology and Business University (BTBU), Beijing, 100048 China

## Abstract

Shanxi aged vinegar (SAV), one of the famous Chinese vinegars, is produced by multispecies solid-state fermentation in which the acetic acid fermentation stage (AAF) is especially important. However, how bacterial succession and their metabolites change along with the different stages of AAF is still poorly understood. In this study, we investigated the dynamic bacterial succession and flavor formation in three batches of SAV using high-throughput sequencing and metabolomics approaches. It is interesting to find that AAF can be divided into three stages based on its bacterial community succession (early stage, days 0–4; medium stage, days 5–21; and later stage, days 22–26). *Pantoea*, *Pediococcus*, *Lactococcus* and *Rhizobium* played an important role in the early stage; *Lactobacillus* was dominant in the medium stage (67.72%); and *Acetobacter*, *Komagataeibacter* and *Kroppenstedtia* were the key bacteria in the later stage. A total of seven organic acids and 42 volatile constituents (esters, alcohol, ketones and aldehydes) were detected during the AAF. Spearman correlation analysis showed a significant correlation between the bacterial community and these flavor metabolites during the AAF of the SAV. This is the first report to explore the relationships between volatile flavor metabolites and bacterial community succession by a three-staged method and provide theoretical support for a flavor formation mechanism in traditional SAV.

## Introduction

Shanxi aged vinegar (SAV) is one of the traditional Chinese rice vinegars and is famous for its complex and pleasant aroma. The history of SAV can be traced back thousands of years^1,2^. SAV has attracted considerable interest because it is considered to be both a conventional and a functional food^3^. Recent evidence suggests that it has several types of therapeutic effects, such as antioxidant, hypotensive, hypoglycemic and cholesterol-lowering activities^4–6^. While the original production of these effects may have been unintentional, SAV is now indispensable due to its abundance of culinary and medicinal benefits.

SAV is traditionally produced from sorghum and other cereals by spontaneous solid-state fermentation via three major steps: starch saccharification, alcohol fermentation (AF) and acetic acid fermentation (AAF)^7,8^. Among these three steps, the AAF is thought to be the most complicated and critical in the formation of flavor metabolites and the accumulation of acetic acid^9,10^. During the AAF process, the alcoholic substances produced by AF are mixed with wheat bran, millet chaff and a small quantity of old *Pei* (acetic acid fermented product from the last batch used as vinegar seed) and followed by fermentation for approximately 20–30 days, stirring every morning to increase the oxygen content^11,12^. During the final step, NaCl is added to the mixture to inhibit the growth of *Acetobacter*. The characteristic aromatic components formed during AAF include organic acids, volatile compounds and amino acids^13,14^. It has been reported that 19 volatile compounds and 87 kinds of nonvolatile metabolites that are considered to be aroma-active compounds have been identified in SAV by using GC-MS^15,16^. Numerous studies suggest that the diverse volatile or nonvolatile compounds in SAV resulted from the bacterial activity during this period^8,10^. The rapid development in culture-independent techniques including denaturing gradient gel electrophoresis (DGGE)^17–19^ and high-throughput sequencing^20^ led to a deep understanding of the dynamics, diversity and function of microbial community succession in vinegar *Pei* (solid-state vinegar culture) during the AAF process. Four bacterial genera including *Acetobacter*, *Lactobacillus*, *Escherichia* and *Klebsiella* have been identified in the vinegar *Pei* of SAV during the AAF process. It was found that the bacterial diversity increased in the first three days of AAF and then decreased as the AAF proceeded^19^. Similar results have also been reported by Nie *et al*., who found that *Acetobacter* and *Lactobacillus* are the major bacterial genera in SAV during the AAF process. In addition, the results showed that the microbiota and nonvolatile flavor metabolites produced during AF and AAF were strongly correlated^16^. However, the relationship between the volatile metabolites and various bacteria, and which bacteria boosted the accumulation of the major flavor substance during the AAF process, is still unclear.

In this study, the bacterial community succession of vinegar *Pei* collected from three batches during AAF was analyzed by high-throughput sequencing. We identified organic acids and volatile flavor metabolites in each sample by HPLC and GC-MS, respectively. At this point, the acetic acid fermentation was divided into three stages. We aimed to determine the relationships between the dynamic succession of the bacterial community and the formation of flavor metabolites (including volatile substance and organic acids) in the different stages of AAF.

## Results

### Composition and changes of bacterial succession in vinegar *Pei* during AAF

**Figure 1:**
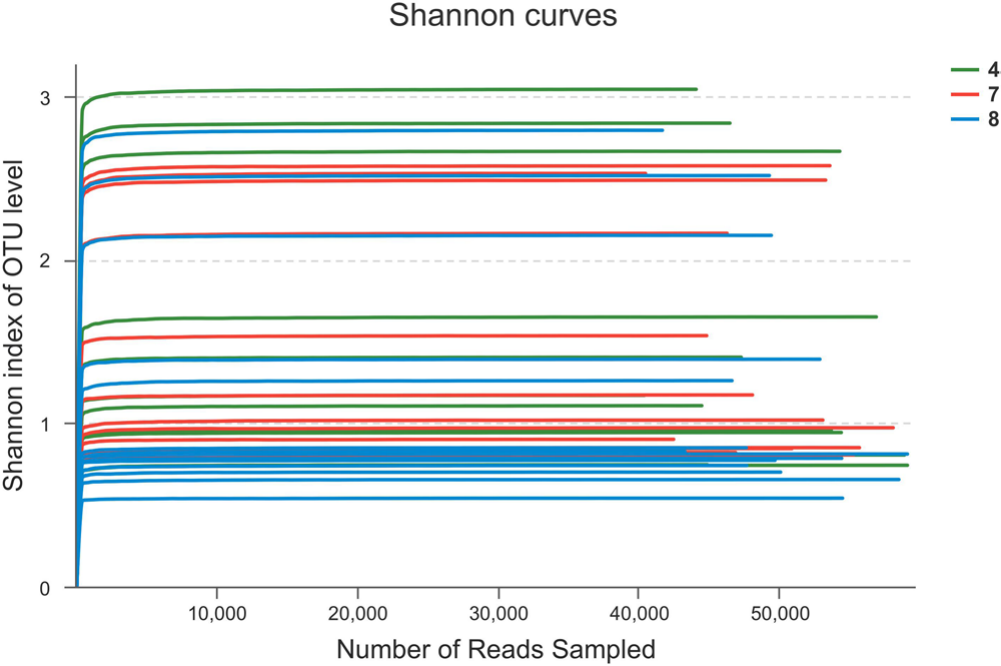
Shannon diversity curves of vinegar *Pei* during the AAF; 4, 7, and 8 represent different fermentation pools.

**Figure 2:**
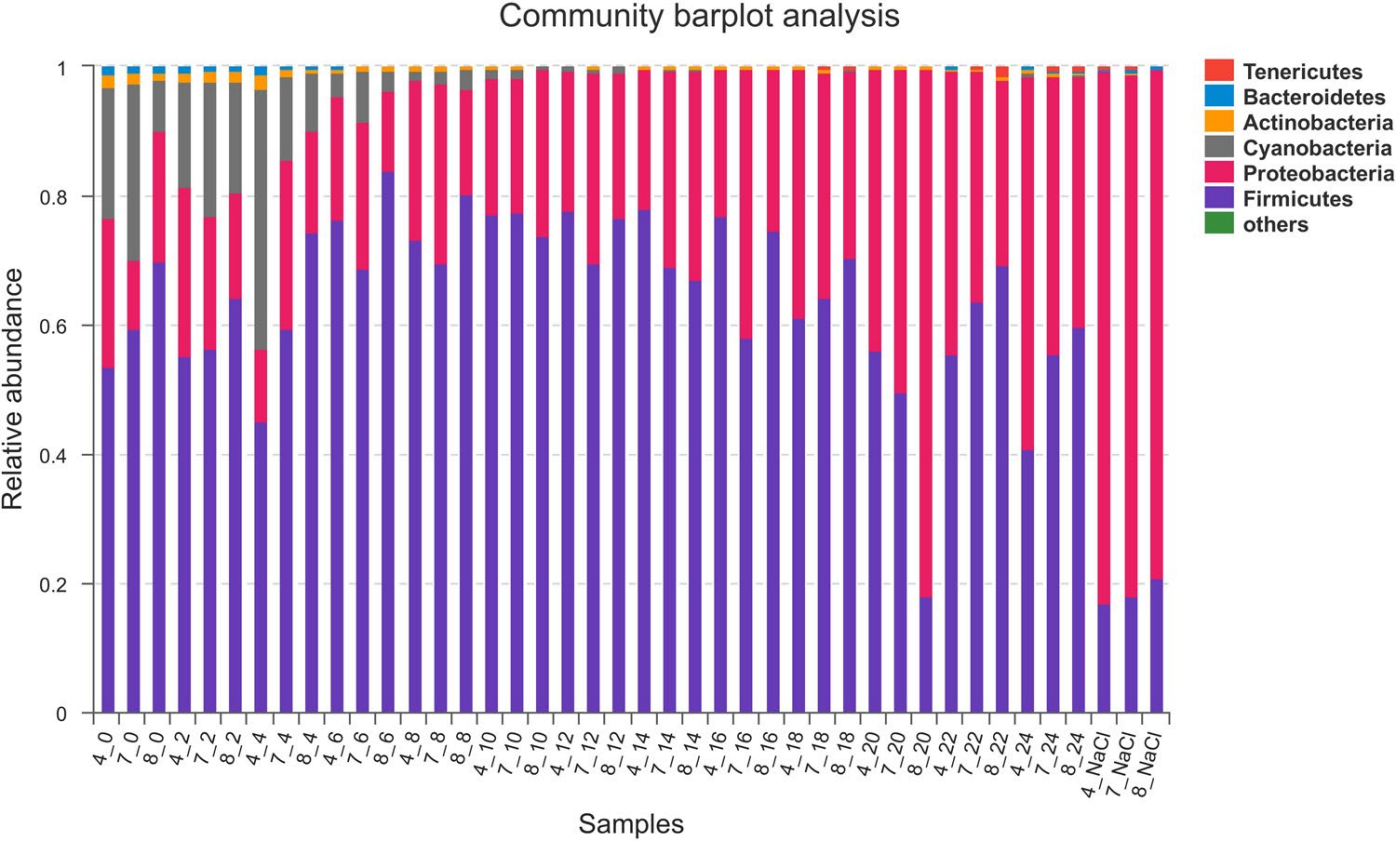
Bacterial community compositions of vinegar *Pei* during the AAF process at the phylum level.

In prokaryotic microbes, high-quality 2,134,087 16S rRNA gene sequences of V3–V4 regions were obtained from 42 *Pei* vinegar samples. For all of the samples, rarefaction and Shannon diversity curves leveled off strongly, indicating that the majority of the diversity was captured in this analysis (Fig. 1). The sequences were classified into 351 operational taxonomic units (OTU) at a 97% similarity level and distributed among six major phyla (relative abundance >1%): *Firmicutes*, *Proteobacteria*, *Tenericutes*, *Bacteroidetes*, *Actinobacteria* and *Cyanobacteria*. *Firmicutes* and *Proteobacteria* were the predominant phyla, together contributing up to 90% of the sequences, while *Cyanobacteria*, *Tenericutes*, *Bacteroidetes*, *Actinobacteria* only accounted for 2%–40% of the sequences (Fig. 2). The abundance of *Firmicutes* decreased rapidly in the samples collected after the NaCl was added on the last day (<20%). In contrast, the proportion of *Proteobacteria* was significantly higher in 4_NaCl, 7_NaCl and 8_NaCl compared with the others. *Cyanobacteria* decreased gradually and finally disappeared on the 12th day.

**Figure 3:**
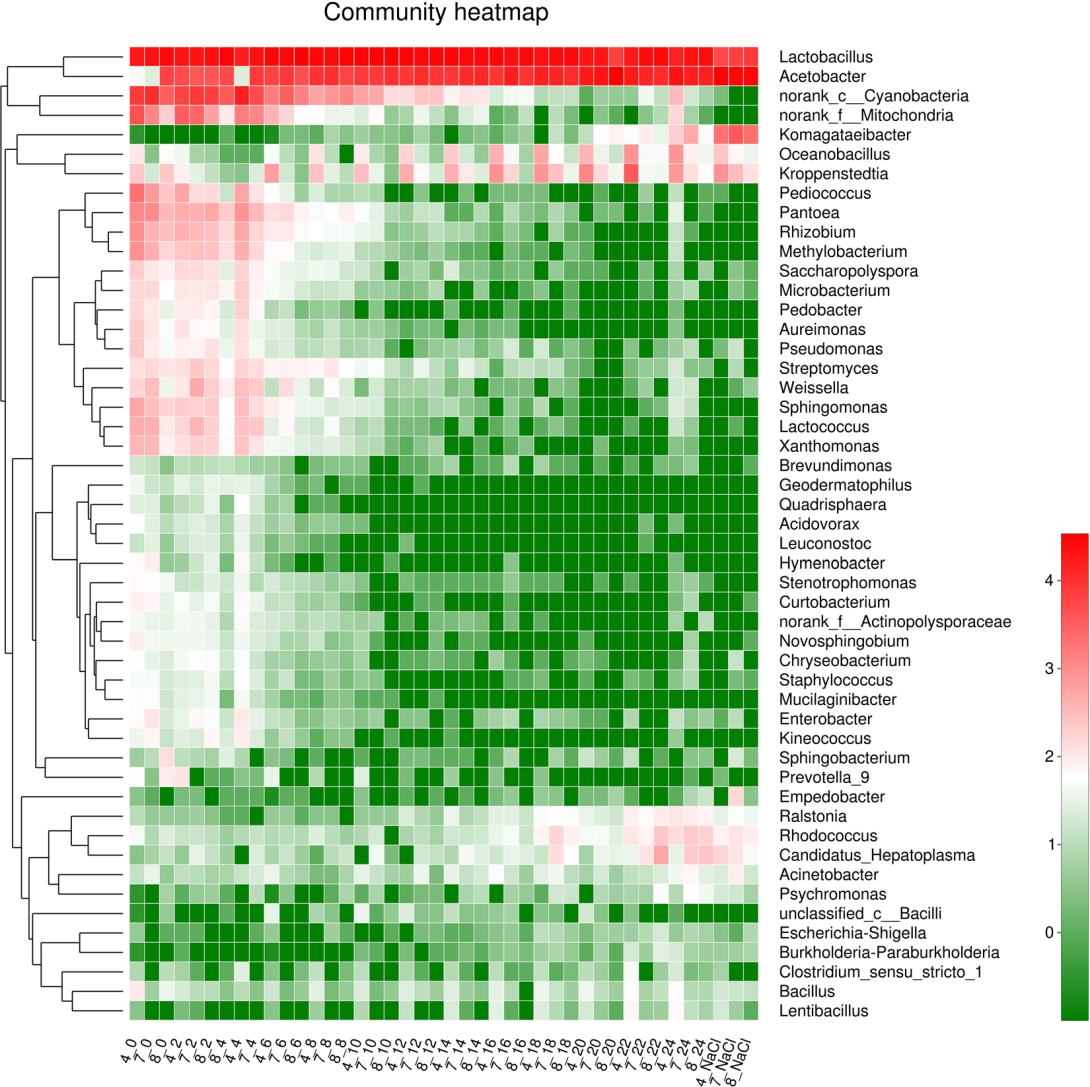
Dynamic changes of the bacterial community abundance in vinegar *Pei* during the AAF process. Different colors represent the relative abundance of bacteria.

The use of RDP classifier Bayesian algorithm analysis allowed bacterial identification at the genus level (Fig. 3). The results showed that the abundance of dominant bacteria differed substantially during the AAF process. The relative abundance of *Lactobacillus* and *Acetobacter* was greater than that of the other bacteria during the AAF. At the beginning of the AAF, *Lactobacillus* was the predominant bacterium, and then decreased during the fermentation process, while *Acetobacter* was highly enriched throughout the AAF and constituted the most dominant genus at the end of the AAF. In addition, *Rhizobium*, *Methylobacterium*, *Pantoea*, *Pseudomonas*, *Streptomyces*, *Lactococcus*, *Xanthomonas* and *Saccharopolyspora* were primarily observed in vinegar *Pei* collected during the first four days and then decreased as the fermentation (AAF) proceeded. *Komagataeibacter* was detected on the 6th day and dramatically increased as the fermentation (AAF) proceeded. *Pedobacter* and *Aureimonas* in the vinegar *Pei* gradually decreased during fermentation and finally disappeared in samples obtained on the 22th day, while *Rhodococcus* and *Candidatus_Hepatoplasma* increased slightly. The abundance of *Bacillus* and *Ralstonia* were relatively stable during the entire AAF process.

### Comparison of bacterial community composition at the different stages of the AAF

**Figure 4:**
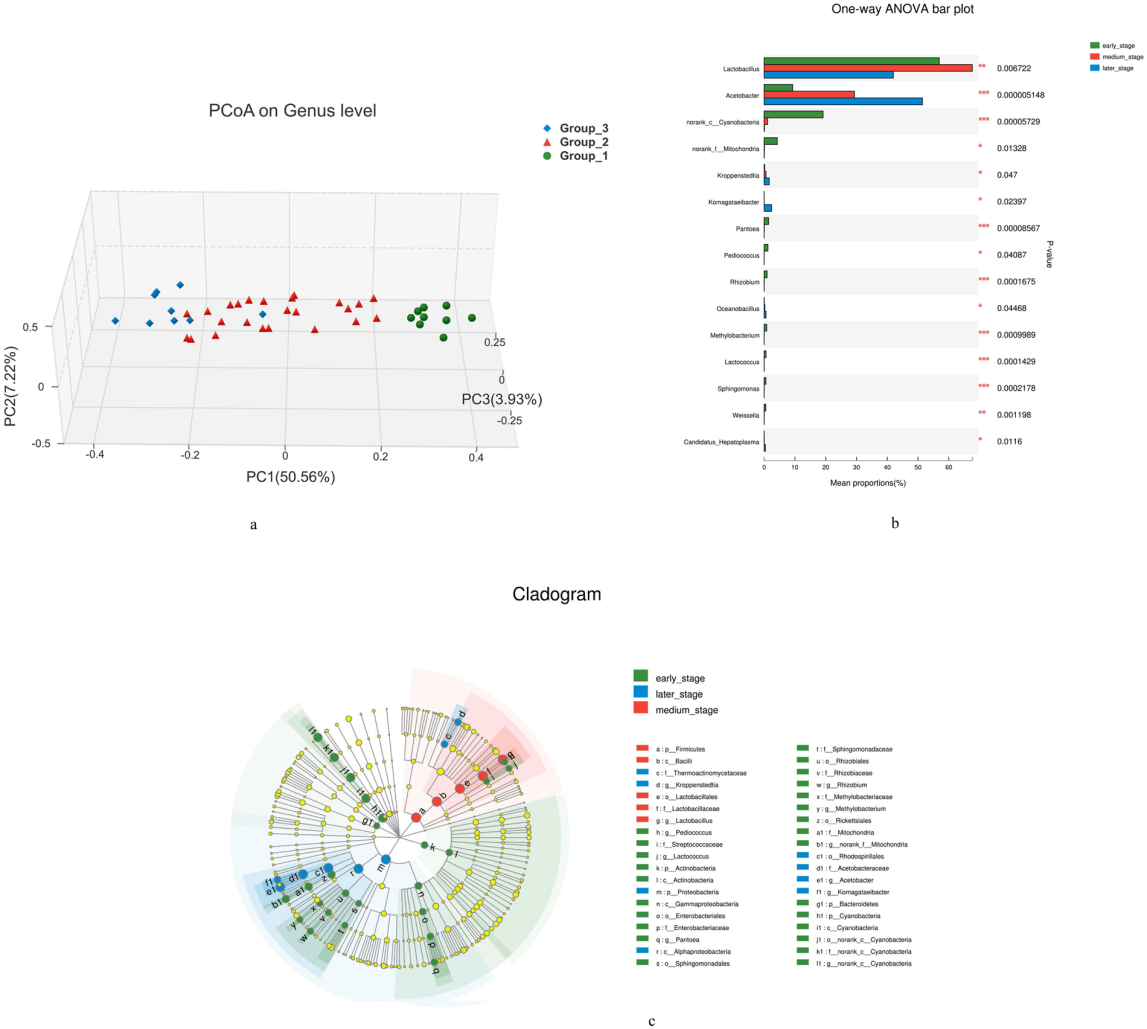
Comparison of bacterial succession among the different stages of the AAF process. (**a**) Principal coordinates analysis to assess the differences of bacterial communities between the three stages of AAF. (**b**) One-way ANOVA showing the difference in bacterial richness between the three stages of AAF process. (**c**) LEfSe showing the dominant bacteria in each stage of AAF.

On the basis of the dynamic changes in the microbial community during the AAF (Figs 2 and 3), the fermentation process could be divided into three fermentation stages: group 1 (samples collected in the first 4 days of AAF) was the earlier fermentation stage; group 2 (samples collected from 6th day to 20th day of AAF) and group 3 (samples collected from the 22th day to the 26th day of AAF) were in the medium and later stages of the fermentation, respectively. The distance between sample points in the PCoA diagram was used to evaluate the similarity of the bacterial profile. It was observed that the samples from the same fermentation stage grouped tightly. A more clear discrimination was exhibited between the different fermentation stages in the first direction (PC1), which accounted for 50.56% of the variability (Fig. 4a).

The Spearman correlation and linear discriminate analysis effect size (LEfSe) identified potential discriminating microorganisms between the three fermentation stages. It was observed that *Lactobacillus*, *Acetobacter*, *Komagataeibacter*, *Pantoea*, *Rhizobium*, *Methylobacterium*, *Lactococcus* and *Candidatus_Hepatoplasma* had statistically significant (*p* < 0.05) differences among the three stages that were consistent with the Alpha-diversity analysis (Fig. 4b). In the early stage, the abundance of *Pantoea*, *Pediococcus*, *Lactococcus* and *Rhizobium* was significantly higher than those in the other two stages. In the medium stages, *Lactobacillus* was the dominant type of bacterium that accounted for 67.62% of the total bacteria. In the later stage, *Lactobacillus* decreased remarkably, while *Acetobacter*, *Komagataeibacter* and *Kroppenstedtia* significantly increased. *Acetobacter*, *Komagataeibacter* and *Kroppenstedtia* prevailed during the later stage (Fig. 4c).

### Analysis of the flavor metabolites and their correlation with the bacteria

In this study, a total of 49 kinds of flavor compounds were detected in vinegar *Pei* during the AAF of SAV (Table 1). These major compounds included seven organic acids and 42 volatile metabolites consisting of seven alcohols, 26 esters, five aldehydes and four ketones. Overall, the microbial diversity and metabolism changed during the AAF, and these metabolites participated in many chemical reactions that caused the varieties of flavor compounds in this period. The correlation Heatmap diagram was used to assess the correlation between microbial classification and environmental variables by the Spearman correlation test.

**Table 1:** Flavor compounds identified in vinegar *Pei* samples.

Code	Compounds	Basis of identification^a^	Concentration (mg/kg)		
			Early stage	Medium stage	Later stage
***Organic acids*** **(7)**					
AC1	Acetic acid	MS	3.95 × 10^4^	1.24 × 10^5^	1.75 × 10^5^
AC2	Lactic acid	MS	7.16 × 10^4^	7.41 × 10^4^	5.31 × 10^4^
AC3	Succinic acid	MS	1.73 × 10^4^	5.37 × 10^4^	5.05 × 10^4^
AC4	Citric acid	MS	7.64 × 10^3^	2.41 × 10^4^	3.22 × 10^4^
AC5	Oxalic acid	MS	6.37 × 10^2^	9.50 × 10^2^	1.03 × 10^3^
AC6	Tartaric acid	MS	1.98 × 10^3^	2.83 × 10^3^	3.15 × 10^3^
AC7	α-Ketoglutaric acid	MS	1.31 × 10^3^	1.95 × 10^3^	1.98 × 10^3^
Σ			1.40 × 10^5^	2.81 × 10^5^	3.27 × 10^5^
***Esters*** **(26)**					
E1	Ethyl acetate	MS	10.80	5.86	4.44
E2	Hexanoic acid, ethyl ester	MS	1.33	0.70	0.62
E3	Propanoic acid, 2-hydroxy-, ethyl ester	MS	1.46	0.88	0.30
E4	Acetic acid, 2-phenylethyl ester	MS	0.65	1.66	2.47
E5	Pentanoic acid, 2-hydroxy-4-methyl-,ethyl ester	MS	1.27	1.31	1.08
E6	1-Butanol, 3-methyl-, acetate	MS	1.01	2.69	1.39
E7	Butanedioic acid, diethyl ester	MS	1.26	0.68	0.49
E8	Acetic acid, hexyl ester	MS	0.02	0.03	0.02
E9	Heptanoic acid, ethyl ester	MS	0.21	0.12	0.11
E10	Octanoic acid, ethyl ester	MS	0.69	0.41	0.09
E11	Nonanoic acid, ethyl ester	MS	0.27	0.16	0.12
E12	3-(Methylthio)propanoic acid ethyl ester	MS	0.11	0.09	0.07
E13	Isoamyl lactate	MS	0.13	0.08	0.01
E14	Decanoic acid, ethyl ester	MS	0.25	0.13	0.09
E15	Benzoic acid, ethyl ester	MS	0.16	0.09	0.09
E16	Benzeneacetic acid, ethyl ester	MS	0.72	0.30	0.32
E17	Dodecanoic acid, ethyl ester	MS	0.21	0.13	0.06
E18	Benzenepropanoic acid, ethyl ester	MS	0.05	0.05	0.07
E19	Tetradecanoic acid, ethyl ester	MS	0.24	0.13	0.07
E20	Pentadecanoic acid, ethyl ester	MS	0.04	0.03	ND^b^
E21	Hexadecanoic acid, ethyl ester	MS	1.37	0.84	0.51
E22	Ethyl 9-hexadecenoate	MS	0.04	0.02	ND
E23	(E)-9-Octadecenoic acid ethyl ester	MS	0.32	0.21	0.17
E24	9,12-Octadecadienoic acid, ethyl ester	MS	0.54	0.36	0.21
E25	Dibutyl phthalate	MS	0.01	0.02	ND
E26	[1,1′-Bicyclopropyl]-2-octanoic acid,2′-hexyl-, methyl ester	MS	ND	ND	0.01
Σ			23.16	16.99	12.80
***alcohols*** **(7)**					
AL1	Ethanol	MS	12.41	6.41	1.93
AL2	3-methyl-1-butanol	MS	3.33	2.11	1.21
AL3	Phenylethyl alcohol	MS	4.32	3.12	3.26
AL4	1-Hexanol	MS	0.19	0.02	ND
AL5	2-methyl-1-Hexadecanol	MS	0.01	0.02	0.03
AL6	[S-(R*,R*)]-2,3-Butanediol	MS	ND	0.11	0.12
AL7	1-Propanol, 3-(methylthio)-	MS	0.10	0.01	ND
Σ			20.36	11.80	6.54
***ketones*** **(4)**					
K1	Acetoin	MS	0.48	0.87	1.89
K2	2-Octanone	MS	0.77	0.15	0.27
K3	3-Acetoxy-2-butanone	MS	ND	0.08	0.28
K4	Acetophenone	MS	0.02	0.01	0.01
Σ			1.27	1.11	2.46
***aldehydes*** **(5)**					
ALD1	Furfural	MS	ND	0.40	1.04
ALD2	Benzaldehyde	MS	0.28	1.06	1.43
ALD3	2(3 H)-Furanone, dihydro-5-pentyl-	MS	0.19	0.21	0.34
ALD4	Benzeneacetaldehyde	MS	0.06	0.08	0.09
ALD5	1H-Indene-4-carboxaldehyde, 2,3-dihydro-	MS	0.04	0.05	0.11
Σ			0.57	1.80	3.02

^a^MS, compounds were identified by MS spectra.

^b^ND, Not Detected.

**Figure 5:**
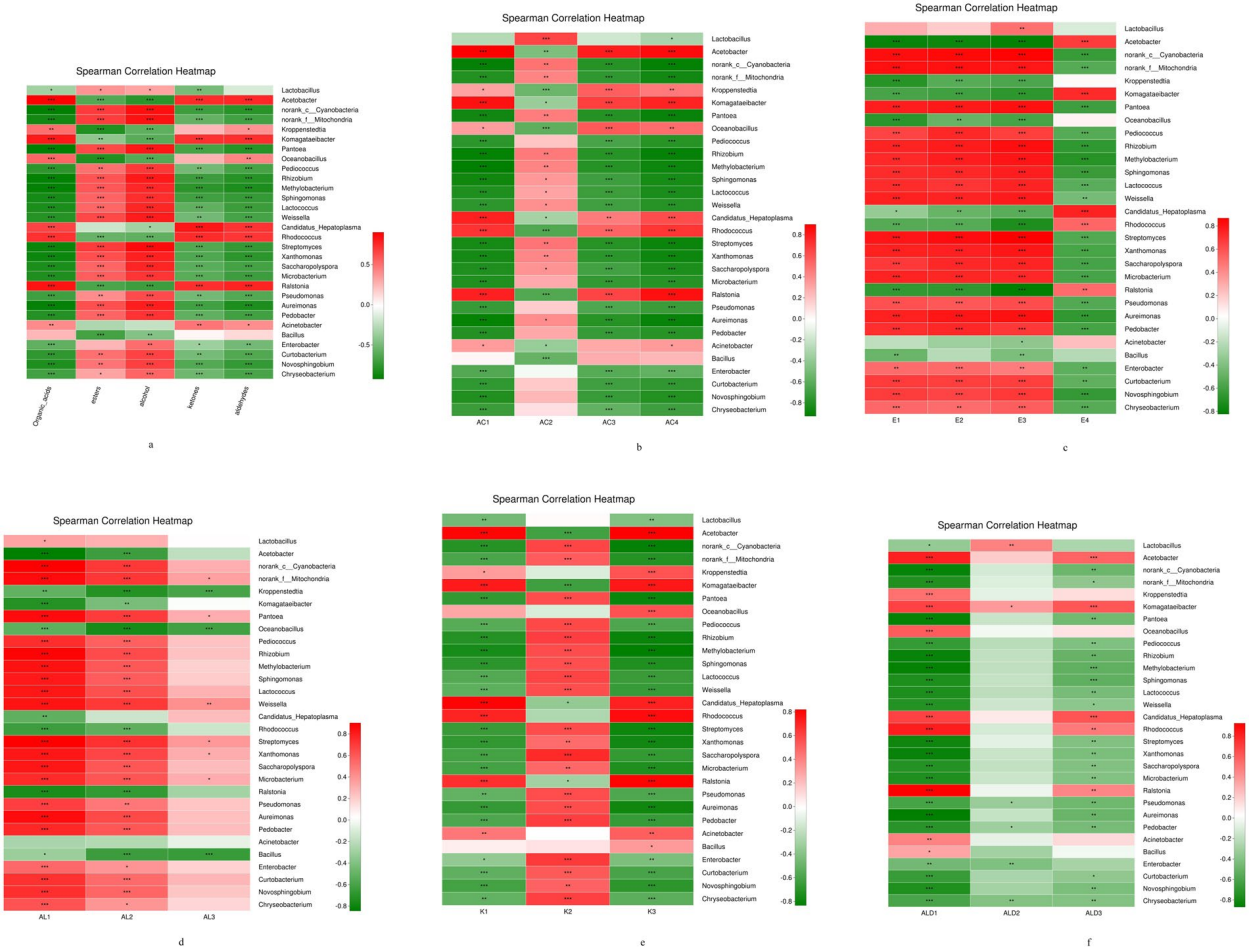
Analyses of flavor metabolites and their correlation to the bacteria. The red color represents the positive correlation and the green color represents the negative correlation. (**a**) Correlations between bacterial community and flavor metabolites. *Represents the significance of relationships, *0.01 < *p* ≤ 0.05, **0.001 < *p* ≤ 0.01, ***p < 0.001 (**b**) Correlations between the dominant bacteria and the major organic acids. (**c**) Correlations between the dominant bacteria and the major esters. (**d**) Correlations between the dominant bacteria and the major alcohols. (**e**) Correlations between the dominant bacteria and the major aldehydes. (**f**) Correlations between the dominant bacteria and the major ketones.

The compositions of organic acids (acetic acid, lactic acid, succinic acid, citric acid, oxalic acid, tartaric acid and α-ketoglutaric acid) were detected by HPLC. Acetic acid, lactic acid, succinic acid and citric acid were the primary organic acids in vinegar *Pei* accounting for more than 98% of the total contents of the seven organic acids detected. The amount of lactic acid was augmented slightly in the medium stage (74.1 g/kg dry *Pei*) and then reduced to 53.1 g/kg dry *Pei*, while the content of acetic acid increased rapidly up to 175 g/kg dry *Pei* by the end of the AAF. The data showed the sustained growth tendency of succinic acid and citric acid during the AAF. As seen from Fig. 5a, *Komagataeibacter* and *Acetobacter* had a significant positive correlation with organic acids, especially acetic acid, suggesting that these microorganisms could contribute to the yield of acids. Further analysis (Fig. 5b) showed that *Lactobacillus* was positively correlated with the lactic acid that probably explained why the lactic acid increased during the early and medium stages. The accumulation of acetic acid, succinic acid and citric acid might contributeto the increasingabundance of *Acetobacter*, *Komagataeibacter* and *Kroppenstedtia*.

As important flavor metabolites, esters could improve the aroma of vinegar and were previously identified^21^. Among the 26 esters identified in the vinegar *Pei*, ethyl acetate was the most abundant ester and decreased gradually during stage three as a majority of the esters in the samples. However, the concentrations of acetic acid-2-phenylethyl ester increased during the AAF process and showed a higher positive correlation with *Acetobacter* and *Komagataeibcter*. The concentration of 1-butanol-3-methyl-acetate increased in the medium stage and then decreased in the later stage (Table 1). The results of the correlation analysis (Fig. 5a,c) revealed a close relationship of *Pantoea*, *Pediococcus*, *Rhizobium* and *Lactococcus* with esters in which propanoic acid-2-hydroxy-ethyl ester, hexanoic acid ethyl ester and ethyl acetate were the most significant. The result also indicated that *Lactobacillus* only contributed to the content of the propanoic acid-2-hydroxy-ethyl ester.

Alcohols mainly originated from the alcohol fermentation stage that provided the precursors for the synthesis of organic acids. In this study, seven alcohols were identified, and ethanol accounts for the highest proportion of all the types of alcohol. Interestingly, 2,3-butanediol was not detected in the early stage and was formed during the medium and late stages, while others decreased during this fermentation process. In addition, Fig. 5d indicated that *Pantoea*, *Pediococcus*, *Rhizobium* and *Lactococcus* had a positive correlation with the three major alcohols including ethanol and 3-methyl-1-butanol. In contrast, *Acetobacter*, *Kroppenstedtia* and *Komagataeibacter* showed a negative correlation with ethanol and 3-methyl-1-butanol.

This study identified four ketones and five aldehydes in the vinegar *Pei* of SAV. These metabolites are usually derived from lipid and amino acid degradation^22^ and contribute honey and fruity odors^23^. Among the substances, acetoin was an important contributor to synthesize pyrazines that could be added to the food to develop an aroma^24^. Figure 5a shows *Acetobacter* and *Komagataeibacter* bound with these compounds at the *p* = 0.01 level. In addition, further statistical tests (Fig. 5e,f) revealed that they were positively correlated with the increasing furfural, benzaldehyde, acetoin and 3-acetoxy-2-butanone, while *Pantoea, Pediococcus, Rhizobium* and *Lactococcus* showed the opposite result. In addition, the correlations between *Lactobacillus* and benzaldehyde, as well as between *Rhizobium* and *Lactococcus* and 2-octanone, suggested that they were the predominant contributors to form these constituents.

## Discussion

AAF is responsible for the flavor metabolite formation and organic acid production attributed to microbial succession, in which the bacteria played key roles^9,10^. The structure and composition of the microbial community of SAV had been investigated previously in *daqu*, *jiulao* and *cupei* samples using cultural-dependent and cultural-independent methods^12,16,19,25,26^. However, it was difficult to explore the AAF using traditional pure cultural technique due to the complexity of the bacterial community. For instance, only four bacterial genera including *Acetobacter, Lactobacillus, Escherichia* and *Klebsiella* were detected in the AAF using the DGGE method^27^. With the rapid development of sequencing techniques, it is easier to comprehensively characterize the bacterial community of SAV^16,17,19,20^. In this study, more than 15 genera of bacteria (relative abundance > 1%) were identified in vinegar *Pei* during the AAF fermentation using high-throughput sequencing that was more diverse than those detected by DGGE. In addition, *Lactococcus* and *Kroppenstedtia* are detected as key microorganisms during the AAF of the SAV for the first time in this study. Among the predominant bacteria, *Acetobacter*, *Pantoea* and *Pseudomonas* probably arose from the starter cultures,^28^ and *Lactobacillus*, *Saccharopolyspora*, *Streptomyces* and *Lactococcus* primarily originated from the alcohol mash^16^. *Bacillus* and the other genera might have come from the raw materials. *Acetobacter*, *Lactobacillus* and *Komagataeibacter* were dominant as the key bacteria of the SAV that was confirmed by the previous study^8,16,19,27^. In this study, we identified *Acetobacter* and *Lactobacillus* as the most abundant genera, and the variety of the bacterial community decreased during the AAF of SAV. This result is similar to that of Tianjin Duliu aged vinegar^29^. This result confirms that they have a lot in common with different kinds of vinegar.

In this study, we first used a three-stage classification method (early stage, days 0–4; medium stage, days 5–21; and later stage, days 22–26) based on the microbial assembly to analyze the bacterial community succession during the AAF of SAV. Interestingly, a distinct difference of the bacterial community exhibited among the three stages during AAF of SAV advanced our understanding of the detailed dynamic bacterial succession. This division provided a succession profile of the microbiota during the AAF, which could be used to search for microbial markers characterizing the AAF process and to develop a microbiota-based strategy to monitor the AAF process. In the previous study, Nie *et al*. reported that the bacterial composition was slightly different during the AAF, and the period of AAF only lasted 7 days^16^. However, *Lactobacillus* increased in the medium stage and then decreased gradually in the later stage during this study that differed from the previous report. Nie *et al*. reported that *Lactobacillus* decreased gradually during the whole process of the AAF of SAV, and a similar trend was also observed in Zhenjiang Aroma vinegar (ZAV)^10^. Although SAV and ZAV are both made with a cycle-inoculation technique^8,10,27^, diverse crude material and geographical locations contribute to the diverse compositions and changes of the bacteria. In addition to *Acetobacter*, *Kroppenstedtia* and *Komagataeibacter* were found to be dominant acid-producing bacteria in the later stages of AAF. The main reason could be attributed to the acid tolerance of these microorganisms^30^. The result confirms that the microbial diversity relies not only on the fermentation process but also the fermentation environment. The strategy of grouping helps us to monitor the composition and dynamic succession of microbes during the AAF, and the succession profile of the bacteria in the three stages of AAF provide a theoretical guidance for the production of SAV.

Microbial behavior contributes to the production of flavor metabolites^9,11,16^. More recently, high-throughput sequencing technology was used to investigate the behavior of bacterial in a natural environment^31,32^, but it was difficult to correlate the bacterial behavior with volatile substances during the AAF due to the complexity and diversity of microorganisms and flavors. The three-stage method used in this paper can reduce the amount of post-production data and can be adopted to easily analyze the relationships. The result suggested a high correlation between the dynamics of the bacterial community and the metabolites, which was confirmed by the previous study on SAV and ZAV^9,16,27^. It is notable that several dominant acid-producing microbes exhibited a high correlation not only with the acid but also with the volatile flavors. Generally, *Acetobacter* and *Komagataeibacter* could oxidize ethanol to acetic acid during the AAF^33–35^. Additives (bran and rice husk) and daily manual stirring could provide glucose and oxygen for the growth of aerobic acid-producing microbes^25,36^. Interestingly, we found that *Acetobacter* and *Komagataeibacter* also had vital impacts on the formation of acetic acid-2-phenylethyl ester, acetoin, 3-acetoxy-2-Butanone butanone and furfural during the late stage of AAF. *Lactobacillus* was another dominant bacterial group which played an important role in the AAF^16,21^. In this study, the concentration of lactic acid increased in the medium stage, which corresponded to the dynamic changes of *Lactobacillus*, suggesting that *Lactobacillus* contributed to the lactic acid production. *Lactobacillus* resisted the high concentration of ethanol and decreased its amino acid transport and metabolism under ethanol stress^37^. To our knowledge, most of the published papers focused on the acid-producing function of *Lactobacillus* in vinegar. In contrast, we found that the content of benzaldehyde was gradually augmented with the increasing abundance of *Lactobacillus*. The statistical analysis further suggested that *Lactobacillus* played an important role in the formation of benzaldehyde. The dominant status of *Acetobacter* and *Lactobacillus* and their correlation with organic acids indicated that acetic acid and lactic acid are the main organic acids as described in the previous report^38^. In addition, we also found that *Lactococcus*, *Pantoea*, *Pediococcus* and *Rhizobium* dominance in the early stage represented positive relevant esters including ethyl acetate, hexanoic acid ethyl ester, propanoic acid 2-hydroxy-ethyl ester, ethanol and 3-methyl-1-butanol which suggests that these bacteria participate in esterification. These bacteria were first identified as the key microbes in the early stage of AAF. SAV possesses antioxidant properties primarily due to its total polyphenols and flavonoids^4^, and ketones and aldehydes primarily accumulated in the later stage. Esters that provide an important factor for the aroma of SAV are mainly produced in the early stage. Therefore, the early stage and late stage might be vital periods during the brewing process of SAV.

In conclusion, the AAF process was systematically elaborated in this study. We investigated the correlation between bacterial succession and flavor metabolites including organic acids and volatile substances in each stage of the AAF. This is the first report to explore the relationships between volatile flavor metabolites and bacterial community succession using a three-staged-method and provides the theoretical support for the flavor formation mechanism of traditional SAV.

## Methods

### Sample collection

The vinegar *Pei* of SAV in the process of fermentation was sampled from the branch of the Shanxi Aged Vinegar Group (Dingzhou, China). A sterilized cylinder-shaped sampler was used to collect vinegar *Pei* every other day in process of the AAF from the top to bottom in the middle of three parallel pools (#4, #7, #8, 11.7 m × 1.41 m × 0.9 m) and four corners. Forty-two samples of vinegar *Pei* (4_0, 4_2, 4_4, 4_6, 4_8, 4_10, 4_12, 4_14, 4_16, 4_18, 4_20, 4_22, 4_24, 4_NaCl, 7_0, 7_2, 7_4, 7_6, 7_8, 7_10, 7_12, 7_14, 7_16, 7_18, 7_20, 7_22, 7_24, 7_NaCl, 8_0, 8_2, 8_4, 8_6, 8_8, 8_10, 8_12, 8_14, 8_16, 8_18, 8_20, 8_22, 8_24, 8_NaCl,) were collected for metagenomic sequencing and flavor analyses. The collected vinegar *Pei* samples were mixed thoroughly and divided into two parts stored at 4 °C and −80 °C, respectively, for further analysis.

### Organic acids analyses

The contents of seven organic acids (oxalic acid, lactic acid, acetic acid, succinic acid, tartaric acid, α-ketoglutaric acid and citric acid) of vinegar *Pei* were analyzed by using high performance liquid chromatography (HPLC; 1260 series, Agilent Technologies, Santa Clara, Calif, USA). Each vinegar *Pei* (10 g) was placed in a 250 mL flask and added to 30 mL deionized water. The flask sealed with plastic wrap was shaken at 100 rpm for 2 h on a rotary shaker at room temperature and then filtered through a double layer of filter paper. The water extract of vinegar *Pei* (5 mL) was mixed with 2 mL zinc sulfate solution (300 g L^−1^) and potassium ferrocyanide solution (106 g L^−1^) to precipitate the protein and fixed capacity in a 50 mL volumetric flask standing for 20 min. After filtration by double layer filter paper and a 0.22 μm filter membrane, the extract was purified using Sep-Pak C18 Cartridges (500 mg 3 mL^−1^, Waters, Milford, Massachusetts,USA) for HPLC analysis. The reversed-phase separation of organic acid substances was performed on a ZORBAX Eclipse Plus C-18 (250 mm × 4.6 mm i.d, 5 μm particle size) at 30 °C, and ultraviolet detection (UVD) was performed at 210 nm. The mobile phase of HPLC was NaH_2_PO_4_ (20 mmol L^−1^, pH 7.2) and the flow rate was 0.9 mL/min. The injection column was 10 μL, and it was parsed for 15 min for each sample^21^.

### Volatile flavor metabolite analyses

The compositions of volatile metabolites in vinegar *Pei* were determined using headspace solid-phase microextraction/gas chromatography-mass spectrometry (HS-SPME/GC-MS)(Trace MS/GC; Thermo Quest Finnigan Co. (Silicon Valley, CA, USA). Each vinegar *Pei* (2 g) was mixed with 5 mL saturated sodium chloride solution and 5 μL 2-octanol solution at 2.1 mg mL^−1^ in methanol as an internal standard in a 15 mL gas chromatography vial. Then, the vial was shaken slightly and equilibrated for 10 min at 40 °C in an incubation furnace, and the volatile metabolites were adsorbed by SPME fiber (50/30 µm divinylbenzene/carboxen on polydimethylsiloxane, DVB/CAR on PDMS (SupelCo Co., Bellefonte, PA, USA) for 40 min at 40 °C. The fiber was inserted into the injection port for 5 min at 250 °C to desorb the adsorbate for mass spectral analysis.

The volatile metabolites of vinegar *Pei* were analyzed using GC-MS as described by Yu *et al*.^39^ Separations were performed using a DB-WAX capillary column (30 m × 0.320 mm i.d, 0.25 μm film thickness). GC-MS conditions were performed as follows: the carrier gas was helium at a constant rate of 1.0 mL L^−1^, and the mode of injection was split-less. The initial temperature of the oven was 32 °C, and it was then heated to 120 °C at a rate of 3 °C/min, and finally 10 °C/min up to 250 °C that was held for 10 min. The temperature of the nozzle and ion source were set to 280 °C and 230 °C, respectively. The electronic impact energy was 70 eV, and the mass spectral scanning range was 18 m/z to 500 m/z. NIST 05a library (Finnigan Co., USA) was used to identify the volatile metabolites by comparing the mass spectral data. Quantification analysis of each compound was conducted by comparing its peak areas with that of 2-octanol in the spectrogram.

### Genomic DNA extraction, PCR amplification and pyrosequencing

Microbial DNA was extracted from vinegar *Pei* using an E.Z.A.N.® soil DNA Kit (Omega Bio-tek, Norcross, GA, USA) according to the manufacturer’s instructions. *Pei* (500 mg) was mixed with 978 μL sodium phosphate buffer and 122 μL MT buffer in a lysing Matrix E tube, and then centrifuged at 14,000 × g for 5–10 min to pellet the debris. The supernatants were mixed with 250 μL Protein Precipitation Solution (PPS) by shaking the tube by hand 10 times. After shaking, the supernatants from an additional centrifugation were transferred to a clean 15 mL tube; 1.0 mL Binding Matrix suspension was added to bind the DNA, stabilized for 3 min to allow settling of silica matrix, resuspended in Binding Matrix after discarding 500 μL supernatants, and the remaining mixture was transferred to a SPIN Filter. The supernatants collected after centrifugation at 14,000 × *g* for 1 min two times were mixed with 500 μL prepared SEWS-M and gently resuspended using the force of the liquid from the pipet tip and centrifuged as before. After centrifugation a second time at 14,000 × *g* for 2 min, the SPIN Filter was placed for 5 min at room temperature to dry out the matrix of the residual wash solution. DNA was collected by resuspending the Binding Matrix in 50 μL DES and centrifuging at 14,000 × *g* for 1 min. The final DNA purity was determined at A260/A280, and the DNA quality was evaluated by 1% agarose gel electrophoresis under ultraviolet light. All of the extracted DNA was ready for PCR and further experiments.

The V3–V4 hypervariable regions of the bacterial 16S rRNA genes were amplified with primers 338 F (5′-ACTCCTACGGGAGGCAGCAG-3′) and 806 R (5′-GGACTACHVGGGTWTCTAAT-3′) using a thermocycler PCR system (GeneAmp 9700, ABI, USA). The PCR reactions were conducted using the following program: 3 min denaturation at 95 °C, 30 cycles 30 s at 95 °C, 30 s annealing at 55 °C, and 45 s elongation at 72 °C, and a final extension at 72 °C for 10 min. PCR reactions were performed in triplicate in 20 μL mixtures containing 4 μL 5 × FastPfu Buffer, 2 μL 2.5 mM dNTPs, 0.8 μL each primer (5 μM), 0.4 μL FastPfu Polymerase and 10 ng template DNA. The resulted PCR products were extracted from a 2% agarose gel.

### Processing of sequencing data

Raw FASTQ files were de-multiplexed, quality-filtered using Trimmomatic and merged using FLASH. Operational taxonomic units (OTUs) were clustered with 97% similarity cutoff using UPARSE (version7.1 http://drive5.com/uparse/), and chimeric sequences were identified and removed using UCHIME. The taxonomy of each 16S rRNA gene sequence was analyzed using an RDP Classifier algorithm (http://rdp.cme.msu.edu/) against the Silva (Release128 http://www.arb-silva.de) 16S rRNA database using a confidence threshold of 70%.

Alpha-diversity was used to reflect the community diversity and richness using the Chao1 richness estimator and Simpson diversity index. Differences and similarities between the samples were reflected by Beta-diversity that was calculated using the Hellinger distance between samples for bacterial 16S rRNA reads. The relationships between samples were determined by unconstrained dimensionality reduction analysis and mapping through distance matrices known as principal coordinates analysis (PCoA). A one-way analysis of variance (ANOVA) was used to determine whether there were any significant differences between the sample groups of the different periods of vinegar fermentation, and the bacteria with significant differences in each group were determined using LDA EffectSize (LEfSe analysis). The correlation between the microbial classification and environmental variables was explored using the Spearman correlation coefficient, and the thermography could intuitively show the distance matrix.
